# Sex differences in the association of childhood sexual abuse severity with premarital sex among Chinese college students in Luzhou, China

**DOI:** 10.1186/s12889-024-17767-9

**Published:** 2024-01-24

**Authors:** Zhang Rong, He Jing, Yang Lin, Cao Rongzhe, Liao Maoxu, Lin Xin, Zhou Ping

**Affiliations:** 1 School of Public Health, Southwest Medical University, 646000 Luzhou, China; 2 School of Clinical Medicine, Southwest Medical University, 646000 Luzhou, China; 3 Information and Education Technology Center, Southwest Medical University, 646000 Luzhou, China; 4 School of Nursing, Southwest Medical University, 646000 Luzhou, China; 5 Department of Radiology, The Affiliated Hospital, Southwest Medical University, No. 25, TaiPing Street, Jiangyang District, 646000 Luzhou, China

**Keywords:** Child sexual abuse (CSA), Premarital sex, College students, China

## Abstract

**Purpose:**

The adverse health consequences of premarital sex and childhood sexual abuse (CSA) are both global public health problems. Based on a random sample of college students from a Chinese city, this study investigated the relationship between CSA severity and premarital sex among students, focusing on sex differences.

**Methods:**

A total of 2722 college students from 4 schools in Luzhou were recruited by multistage random sampling. Self-administered questionnaires were used to measure CSA experiences and premarital sex. Binary logistic regression analyses were conducted to analyse the relationship between CSA and premarital sex.

**Results:**

The prevalence of CSA was 9.39%, and that of mild, moderate and severe CSA was 4.04%, 2.90% and 2.46%, respectively. The premarital sex reporting rate was 22.42%. After adjusting for confounding variables, CSA was positively associated with premarital sex. Notably, a cumulative effect of CSA on premarital sex was observed among students. Further stratification analyses showed that males who experienced CSA had a higher premarital sex rate than females, and this sex difference was also observed among students with different CSA severities.

**Conclusion:**

CSA and its severity were associated with premarital sex among college students. Furthermore, this association was stronger for males than females. Therefore, it is important to emphasize CSA prevention, especially for boys. These findings can promote understanding of the effects of CSA on premarital sex, and CSA prevention and intervention strategies should consider CSA severity and sex differences.

## Background

In most countries, the shift towards later marriage in recent decades has led to an increase in premarital sex, making sexual health in relation to premarital sex an important public health issue [1]. Contemporary Chinese college students have open attitudes towards premarital sexual behaviour, and premarital sex is common among college students [2]. The premarital sex rate reported in China from 2011 to 2019 ranged from 13.36 to 30.4%, accompanied by an increasing trend year by year [3–5]. Limited precollege sex education and the increasingly open sexual attitudes [6] of college students regarding one-night stands, sex without condoms, multiple sex partners, and other risky premarital sexual behaviours have resulted in a high incidence of unwanted pregnancies, abortion, HIV and other sexually transmitted infections, which are a potential source of concern for the sexual physiology and psychosexual health of college students [7–9]. Chinese university students who are sexually active have a high incidence of premarital sex, and the average age of these students is only 20.14 ± 2.98 years [10]. The factors influencing premarital sex are numerous and complex, and they include peer sexual behaviour, media messages, family environmental factors, parental and personal attitudes towards premarital sexual behaviour, etc. [11–13]., Among these factors, the influence of adverse childhood experiences, especially childhood sexual abuse (CSA) [14, 15], on premarital sexual behaviour has attracted the attention of researchers.

CSA, as a common adverse childhood experience, is a well-known risk factor for adolescent psychology and behavioural health problems. Under the influence of China’s traditional Confucian ideology and culture, adolescents who experience CSA are labelled as having destroyed chastity (for females) and integrity (for both males and females), leading to CSA being a taboo subject for adolescents who experience it and their parents [16]. However, in China, CSA is common and highly prevalent, with several studies in recent years reporting CSA rates ranging from 12% to 27% [17–20]. As a result, the experience of CSA may increase the likelihood of risky sexual behaviours and adverse reproductive health outcomes in early adulthood [18], which may also lead to long-term adverse effects [21, 22]. Studies in the United States have shown that adolescents with adverse childhood experiences are at higher risk of early sexual debut [23]. Earlier findings from our research group also showed that middle school students who had experienced CSA were more likely to have open attitudes about premarital sex [24]. However, there are relatively few studies in China on whether CSA experiences directly affect individuals’ premarital sexual behaviour.

The effects of CSA experiences on health vary from person to person and include low self-esteem, anxiety, or panic disorder in mild cases; depression or nonsuicidal self-injury in moderate cases; and suicide or even death in severe cases [17, 25, 26]. This may be related to the individual and the exposure to different levels of CSA severity. In addition, a large study in China suggested a dose‒response relationship between the types of child abuse experienced and mental health [27]. It is evident that adverse childhood experiences are associated with mental health and are also likely to influence individual behaviour. We are interested in whether adolescents with experiences of different severities of CSA behave differently in terms of premarital sex. To fully explore the relationship between CSA severity and premarital sex among college students, this study referred to a definition of CSA similar to that in our earlier article, which defined CSA as sexual harassment, sexual molestation, and sexual assault [24].

Men engage in significantly more premarital sex than women [28]. Furthermore, the CSA rate among females is twice that among males [29], and the nature and extent of child maltreatment differs between the sexes [30], as do the resulting negative health outcomes [26]. If different levels of CSA severity affect premarital sex, are there sex differences in the effects? Only one study discovered that sex modifies the effects of CSA on health, implying that the effect of child maltreatment on the risk of depressive symptoms in college students is moderated by sex, with a greater effect in females than males [27]. However, it is unclear whether sex modifies the effect of CSA severity on premarital sex.

To our knowledge, there is a dearth of studies investigating the associations between the levels of CSA severity and premarital sex in college students, and no study in China has assessed the moderating effect of sex on these associations. Therefore, we conducted this cross-sectional study among college students to explore the effects of different levels of CSA severity on premarital sex behaviour, as well as the moderating role of sex on these effects. These findings will have implications for the prevention of CSA and the promotion of reproductive health in the college population.

## Methods

### Study design and sample

Data were obtained from a school-based cross-sectional survey conducted in Luzhou City. The study population was selected using a stratified cluster sampling method based on the composition ratio of 5:4:2:2 of the total number of students in the four colleges and universities (a medical school, a police officer school, and two vocational and technical schools) in Luzhou. In the first stage, 4–6 majors were selected from each grade in the 4 schools, for a total of 28 majors. In the second stage, 2–4 classes were selected from each grade level of the sampled majors, and all students in the selected classes participated in the survey. From January-April 2022, uniformly trained and qualified investigators used classroom time to fully explain the purpose, meaning and confidentiality of the study. Since a web-based survey format can avoid response bias for sensitive questions, respondents completed the questionnaire online through the Questionnaire Star platform. During the questionnaire development process, attention was given to questionnaire integrity, confidentiality, etc. Ultimately, a total of 2978 students participated in this study, with a response rate of 91.40%, and a total of 2722 subjects were included in the study. The study was approved by the Ethics Committee of the Affiliated Hospital of Southwest Medical University (No. KY2022240). Written informed consent was obtained from the participants or their guardians.

### Measures

#### Sociodemographic variables

Sociodemographic variables included sex (male = 1, female = 2), educational level (junior college = 1, undergraduate college = 2), major, grade (1st to 5th), only-child family status, and parental education level. Sex was defined as the biological sex of the student. Parental educational level was based on the highest level of education parents had completed.

#### Premarital sex

For the survey, premarital sex was defined as follows: any instance of engagement in oral sex, anal sex, vaginal intercourse, genital rubbing or other sexual acts. Premarital sex was operationalized with the following question: “As of now, have you ever had sex (including oral, anal, and vaginal sex or genital friction)?”. The response options were “yes” or “no”.

#### Sex-related knowledge

The level of sex-related knowledge was measured with 10 questions (e.g., Can the emergency contraceptive pill be used more than once during a menstrual cycle?). For each question, a score of 1 point was given for a correct answer and 0 points were given for an incorrect answer or do not know response, and the total score ranged from 0 to 10 points. The higher the total score was, the better the knowledge of safe sex behaviour. The standardized Cronbach’s alpha coefficient for this scale was 0.80.

#### Attitudes towards premarital sex

Attitudes towards premarital sex were evaluated using a modified Guttman scale to assess individual attitudes towards premarital sex occurring in different situations (with strangers, boyfriends/girlfriends, or fiancés/wives) [31]. The scale had a total of 6 items and was scored using a Likert scale with the following response options: 1="Totally disagree”, 2="Somewhat disagree”, 3="Neutral”, 4= “Somewhat agree” and 5= “Completely agree”. The total scale score ranged from 6 to 30, with higher scores indicating more conservative attitudes towards premarital sex. The standardized Cronbach’s alpha coefficient for this scale was 0.87. Data on the attitudes of parents and friends towards premarital sex were also collected (agree = 1, disagree = 2, unclear = 3).

#### Childhood sexual abuse

Based on relevant literature [29], the CSA experience survey asked about any experience of sexual abuse before the age of 18 years and contained the following 11 questions: a) “Did someone make sexual advances to you verbally or through obscene books/images? “; b) “Did someone look at your genitals?”; c) “Did someone expose their genitals in front of you?”; d) “Did someone play with their genitals or masturbate in front of you?”; e) “Did someone touch or fondle your genitals?“; f) “Did someone force you to touch their vulva?”; g) “Did someone deliberately rub their genitals on you? “; h) “Did someone touch your genitals with their mouth? “; i) “Did someone force you to touch their genitals with your mouth? “; j) “Did someone place a foreign object in your vagina/anus? “; and k) “Did someone force you to have sexual or anal intercourse?“. The answer options included “yes” and “no”. If a respondent answered “yes” to any of the above questions, he or she was defined as having experienced CSA. Referring to previous research [24], we classified the above 11 survey items into three categories: a ~ d were considered sexual harassment, e ~ i were considered sexual molestation, and j ~ k were considered sexual assault. We then identified four severity levels of CSA according to the following classification: “none” referred to none of the above categories being experienced; “mild” referred to the occurrence of sexual harassment only; “moderate” referred to the occurrence of sexual molestation with or without sexual harassment; and “severe” referred to the occurrence of sexual assault with or without harassment and molestation. The standardized Cronbach’s alpha coefficient for this scale was 0.91.

### Analysis

EpiData Version 3.1 software (EpiData Association., DNK) and double entry were used to build the database. Subsequently, SPSS Statistics Version 22.0 (IBM, Inc., Armonk, NY) was used for data analysis. The continuous data conforming to a normal distribution are described by ($\stackrel{-}{\varvec{x}}\pm \varvec{s}$) and were analysed by ANOVA; the categorical data are described by percentages and were analysed by the *χ*^2^ test. Demographic variables with *P*<0.10 or that have been widely reported in the literature (i.e., sex, age, education level) were entered into the following binary logistic regression models as covariates. Adjusted covariates included sex, age, education level, parental education level, sex-related knowledge score, attitude towards premarital sex, and parental approval of premarital sex. Binary logistic regression models were performed to assess the associations between CSA and premarital sex, and the adjusted odds ratios (a*OR*s) and 95% confidence intervals (95% *CI*s) were calculated. To determine whether sex moderated the association between CSA severity and premarital sex, the multiplicative interaction was examined by adding the product term of sex and CSA to the logistic regression model and computing the *P* value of the interaction. If these interactions were found to be significantly related to premarital sexual behaviour, sex stratification analyses were carried out to determine whether the effects of CSA on premarital sexual behaviour differed between males and females. All hypothesis tests were two-sided, and *P* < 0.05 was considered statistically significant.

## Results

### Sociodemographic characteristics and their associations with premarital sex

Of the 2722 students, 1121 (41.18%) were male, and 1601 (58.82%) were female; the average age was 19.95 ± 1.45 years. In total, 1368 (50.26%) students were junior college students, and 1354 (49.74%) were undergraduate students. The number of self-reported premarital sex cases was 611, accounting for 22.42% of the students. The rate of CSA experiences was 9.39% (256/2725, 95% *CI*: 8.30-10.49%), including 110 people (4.04%) with mild CSA, 79 people (2.90%) with moderate CSA, and 67 people (2.46%) with severe CSA. The reported premarital sex rate was 22.37% (609/2722, 95% *CI*: 20.81–23.94%). The differences in premarital sex reporting rates among students by sex, education level, grade, only-child family status, parental education levels, parents’ and friends’ attitudes towards premarital sex, and CSA severity were statistically significant (all *P* < 0.05). There were also statistically significant differences in the mean premarital sex attitude scores and sex-related knowledge scores between the groups of students with or without premarital sex experiences (all *P* < 0.05). For details, see Table 1.

**Table 1:** Sociodemographic characteristics and their associations with premarital sex [*n(%)]/(*x ®±s)]

Variable	Total	Premarital sex		χ^2^/F	*P*
		No	Yes		
Sex					
Male	1121(41.18)	758(67.62)	363(32.38)	109.927	< 0.001
Female	1601(58.82)	1355(84.63)	246(15.37)		
Educational level					
Junior college	1368(50.26)	1114(81.43)	254(18.57)	22.938	< 0.001
Undergraduate college	1354(49.74)	999(73.78)	355(26.22)		
Grade					
Junior(grade 1–2)	1216(44.67)	1027(84.46)	189(15.54)	60.033	< 0.001
Senior(≥ grade 3)	1506(55.33)	1085(72.05)	422(27.95)		
Only-child family status					
Yes	793(29.13)	575(72.51)	218(27.49)	16.872	< 0.001
No	1929(70.87)	1538(79.73)	391(20.27)		
Father’s education level					
Primary school or below	725(26.63)	584(80.55)	141(19.45)	14.826	0.002
Middle school	1165(42.80)	919(78.88)	246(21.12)		
High school/vocational college/junior high school	549(20.17)	409(74.5)	140(25.5)		
Undergraduate and above	283(10.40)	201(71.02)	82(28.98)		
Mother’s education level					
Primary school or below	1035(38.02)	826(79.81)	209(20.19)	29.409	< 0.001
Middle school	1046(38.43)	832(79.54)	214(20.46)		
High school/vocational college/junior high school	444(16.31)	329(74.1)	115(25.9)		
Undergraduate and above	197(7.24)	126(63.96)	71(36.04)		
Parents’ approval of premarital sex					
Yes	916(33.65)	608(66.38)	308(33.62)	105.988	< 0.001
No	903(33.17)	773(85.6)	130(14.4)		
Not clear	903(33.17)	732(81.06)	171(18.94)		
Friends’ approval of premarital sex					
Yes	1831(67.27)	1318(71.98)	513(28.02)	106.139	< 0.001
No	386(14.18)	356(92.23)	30(7.77)		
Not clear	505(18.55)	439(86.93)	66(13.07)		
Severity of CSA					
None	2466(90.6)	1954(79.24)	512(20.76)	41.654	< 0.001
Mild	110(4.04)	71(64.55)	39(35.45)		
Moderate	79(2.90)	51(64.56)	28(35.44)		
Severe	67(2.46)	37(55.22)	30(44.78)		
Attitude towards premarital sex		13.98 ± 4.73	17.97 ± 4.52	342.519	**< 0.001**
Sex-related knowledge score		6.42 ± 2.77	7.93 ± 2.14	155.109	**< 0.001**

### Associations between CSA and premarital sex

A logistic regression model was fitted with premarital sex as the response variable and other variables, such as CSA or CSA severity, as the independent variables. The results (Model 1) suggested that after controlling for other confounding factors, students who had experienced CSA reported higher rates of premarital sex than those who had not experienced CSA (a*OR* (95% *CI*): 2.36 (1.75~3.20)). The results (Model 2) suggested that after controlling for other confounding factors, compared with students who had not experienced CSA, those who had experienced mild (a*OR* (95% *CI*): 1.81 (1.15~2.85)), moderate (a*OR* (95% *CI*): 2.18 (1.28~3.72)) and severe (a*OR* (95% *CI*): 3.25 (1.87~5.67)) CSA reported higher rates of premarital sex; that is, the more severe the CSA experience was, the higher the rate of premarital sex reported by the college student (Table 2).

**Table 2:** Associations between CSA severity and premarital sex

Variables	Model 1			Model 2	
	aOR (95% CI)	P		aOR (95% CI)	P
Sex: Male	2.43(1.93 ~ 3.05)	**< 0.001**		2.39(1.91 ~ 3.01)	**< 0.001**
CSA(Ref: No)	2.36(1.75 ~ 3.20)	**< 0.001**		-	-
Severity of CSA(Ref: None)	-				**< 0.001**
Mild	-	-		1.81(1.15 ~ 2.85)	**0.011**
Moderate	-	-		2.18(1.28 ~ 3.72)	**0.004**
Severe	-	-		3.25 (1.87 ~ 5.67)	**< 0.001**

Note:

Both models were conducted by controlling for the following covariates: age, sex, education level, paternal and maternal education level, sex-related knowledge score, attitude towards premarital sex, and parents’ and friends’ approval of premarital sex

### Interaction effects between CSA severity and sex

Additionally, since sex (a*OR* (95% *CI*): 2.43 (1.93~3.05) and 2.39 (1.91~3.01), respectively) was found to be an influencing factor of the premarital sex rate among students in Model 1 and Model 2, sex was considered to possibly moderate the relationship between CSA severity and premarital sex. Logistic regression Model 3 was then fitted again by adding the product of sex and CSA severity to Model 2. In the results of Model 3, the interaction effect was statistically significant (a*OR* (95% *CI*): 0.43 (0.26~0.71)) after controlling for other covariates. Thus, we can deem that the relationship between the severity of CSA and premarital sex differs between the sexes (Table 3).

**Table 3:** Interaction effects between CSA severity and premarital sex

Variables	Model 3	
	aOR (95% CI)	P
Sex: Male	2.14(1.69 ~ 2.71)	**< 0.001**
Severity of CSA (Ref: None)		**0.001**
Mild	7.81(2.91 ~ 21.00)	**< 0.001**
Moderate	52.42 (7.26 ~ 378.58)	**< 0.001**
Severe	351.72(19.05 ~ 6494.15)	**< 0.001**
Sex* Severity of CSA	0.43(0.26 ~ 0.71)	**0.001**

Note:

Model 3 was conducted by controlling for the following covariates: age, education level, paternal and maternal education level, sex-related knowledge score, attitude towards premarital sex, and parents’ and friends’ approval of premarital sex

### Associations between CSA severity and premarital sex, stratified by sex

The results of the analysis of associations between the severity of CSA and premarital sex according to sex stratification are shown in Table 4. In the male population (Model 4), after controlling for other confounders, compared to students who had not experienced CSA, those who had experienced mild (a*OR* (95% *CI*): 5.97 (2.16 ~ 16.48)), moderate (a*OR* (95% *CI*): 8.65 (1.43 ~ 52.19)), and severe (a*OR* (95% *CI*): 14.55 (2.98 ~ 71.02)) CSA all reported higher rates of reported premarital sex, meaning that there was a positive association between CSA severity and premarital sex. However, this relationship was only present in female students who had experienced severe CSA (Model 5) (a*OR* (95% *CI*): 2.15 (1.12~4.13). Furthermore, the relationship between CSA severity and premarital sex was significantly stronger in males than in females.

**Table 4:** Associations between CSA severity and premarital sex, stratified by sex

Variables	Model 4			Model 5	
	aOR (95% CI)	P		aOR (95% CI)	P
Severity of CSA(Ref: None)		**< 0.001**			0.052
Mild	5.97(2.16 ~ 16.48)	**0.001**		1.14(0.64 ~ 2.02)	0.660
Moderate	8.65(1.43 ~ 52.19)	**0.019**		1.69(0.94 ~ 3.06)	0.081
Severe	14.55(2.98 ~ 71.02)	**0.001**		2.15(1.12 ~ 4.13)	**0.021**

Note:

Model 4: male college students

Model 5: female college students

Both models were conducted by controlling for the following covariates: age, education level, paternal and maternal education level, sex-related knowledge score, attitude towards premarital sex, and parents’ and friends’ approval of premarital sex

## Discussion

We found a relationship between CSA experiences and early adult sexual behaviour and a cumulative effect among different severities of CSA experiences and premarital sex, with higher rates of premarital behaviour reported by students who had experienced more severe CSA. Additionally, there was a sex difference in this relationship, with a greater effect in males than females. Therefore, it is important to break gender stereotypes and consider the severity of CSA experiences, especially for protection against and prevention of adverse health consequences of early CSA in boys.

We found that 22.42% of college students had engaged in premarital sex and 9.39% had experienced CSA, with 4.04%, 2.90% and 2.46% experiencing mild, moderate and severe CSA, respectively. The rate of reported premarital sex in this study was higher than the rates in Taiwan [32], Wuhan [33] and India [34], and The rates of CSA in this study were somewhat lower than those reported in other studies [19, 35]. This may be due to differences in study populations, behavioural definitions, and measurement instruments or because CSA rates are indeed declining while the premarital sex rate is rising.

Additionally, this study found that after controlling for other confounding factors, the rate of reported premarital sex was 2.36 times higher among students who had experienced CSA than among those who had not. This is similar to the results of another Chinese study [30], in which adolescents who had experienced CSA were at higher risk of early sexual debut. In addition, we found a cumulative effect of CSA severity on premarital sex, with a*OR* values increasing from 1.81 to 2.18 to 3.25 as CSA severity increased from mild to severe, respectively. In other words, a cumulative effect was found, as the odds of premarital sex increased with the increasing severity of CSA. This finding is similar to a previous study that reported a dose‒response relationship between CSA and mental health problems [27]. One possible reason for this result is that the experience of CSA may affect an individual’s sex-related beliefs and subjective norms. The theory of planned behaviour (TPB) indicates that individual behaviour is influenced by subjective norms [36], and perpetrators are not punished accordingly, individuals who experience CSA may change their subjective norms, such as social morality regarding sexual behaviour, and be more likely to engage in unsafe premarital sex, such as one-night stands or commercial sex. Notably, this effect was more pronounced in individuals who had experienced severe CSA than in those who had experienced moderate and mild CSA. Similar relationships between CSA and murderous behaviour were reported in another study [37]. Official CSA prevention and control organizations are still lacking in China, and school- and family-based sex education is optional. Therefore, the results of this study suggest that among individuals who experience CSA, those who experience moderate to severe CSA should be identified and assessed by interventionists and administrators.

We investigated whether sex plays a moderating role in the relationship of CSA severity with premarital sex. There was a significant sex difference in the relationship between CSA and premarital sex, and this relationship held true after sex stratification. Compared to females who had experienced CSA, males who had experienced CSA reported a higher rate of premarital sex. Similarly, Tesfaye et al. also reported this sex-dependent phenomenon [38]. In addition, a previous study also considered the same trend [39]. Two possible reasons for these findings are as follows. First, cultural differences cause individuals in Eastern cultures to be relatively conservative, while traditional Chinese culture emphasizes female chastity and integrity [12]. In intimate relationships, female victims of CSA may be cautious about having premarital sex due to fear of revealing their past experiences. At the same time, as reported by Xiaoliang Chen et al. [27], the aforementioned reasons may lead to female respondents’ reluctance to disclose their CSA experiences, leading to an underestimation of the relationship between CSA and premarital sex in the female population. In addition, influenced by the division of labour, men hold the initiative and a dominant position in sexual activities [40, 41]. Coupled with the pursuit of ideal masculinity in men, the experience of CSA may have implications for the sexual rights and status corresponding to male roles in traditional Chinese social and cultural contexts [39, 42]. This means that the experience of CSA may expose men to more self-esteem conflicts, and they may try to prove their dominance and initiative by having sex in adult relationships. However, boys are perceived to be at lower risk of experiencing CSA than girls, resulting in the health risks of male CSA being underappreciated. Therefore, it is important to break the gender stereotype to communicate that sexual abuse is harmful to all children and adolescents, and it is especially important to increase protection from sexual abuse for boys.

This study also has several limitations. First, this was a cross-sectional study, so the ability to draw causal inferences and validate underlying mechanisms is limited. Second, recall bias is inevitable when study participants self-report their CSA experiences, but this bias should still be relatively small for those who experienced moderate to severe CSA. In addition, the results of this study represent only the population of college students in Luzhou City, and the extrapolation of the findings is limited by environmental factors such as economic development and education level. A larger sample is also needed to verify the results. Finally, questions regarding both CSA and premarital sex are sensitive, and there may have been instances when the respondents concealed the facts, causing the results to be underestimated. Despite these shortcomings, to our knowledge, the present study is one of the few studies examining the relationship between CSA and premarital sex among Chinese college students and considering the cumulative effects of CSA severity and sex differences.

## Conclusion

This study found higher premarital sex rates among college students. There was a positive relationship between CSA experience and premarital sex and a cumulative effect between premarital sex and CSA severity; that is, the more severe the CSA experience was, the higher the rate of reported premarital sex among college students. There were sex differences in this relationship, which showed a more pronounced relationship among male students who experienced CSA. This study elucidates the effects of CSA on premarital sex in Chinese college students, especially among males, which can contribute to a better understanding of potential sex differences in the relationships among different severities of CSA and premarital sex and provides a scientific basis for the development of sex-specific and severity-specific CSA prevention and intervention approaches, as well as for the promotion of sexual and reproductive health among university students.

## Data Availability

The datasets used during the current study are available from the corresponding author upon reasonable request.

## Abbreviations

CSA: Childhood sexual abuse
CI: Confidence interval
